# Clinical Management of Triple-Class Refractory Multiple Myeloma: A Review of Current Strategies and Emerging Therapies

**DOI:** 10.3390/curroncol29070355

**Published:** 2022-06-23

**Authors:** Margaret E. Stalker, Tomer M. Mark

**Affiliations:** 1 School of Medicine, University of Colorado, Aurora, CO 80045, USA; margaret.stalker@cuanschutz.edu; 2 Department of Medicine, Division of Hematology, University of Colorado, Aurora, CO 80045, USA

**Keywords:** multiple myeloma, bispecific antibodies, chimeric antigen receptor T cells, immunomodulatory agents, proteasome inhibitors, monoclonal antibody

## Abstract

Major progress has been made in the upfront treatment of multiple myeloma, but the disease ultimately relapses and leads to death in the vast majority of those afflicted. New treatment strategies and modalities are necessary to treat myeloma in relapse, particularly in cases of triple-refractory status defined by disease progression during or shortly after treatment with immunomodulatory agents, proteasome inhibitors, and anti-CD38 monoclonal antibody therapy. In this manuscript, we review recent promising developments in the treatment of triple-class refractory myeloma including bispecific antibodies and T cell engagers, chimeric antigen receptor cellular therapies, as well as chemotherapeutics with novel mechanisms of action.

## 1. Introduction

Multiple myeloma (MM) is an incurable malignancy of plasma cells, and the American Cancer Society estimates that there will be approximately 34,470 new MM cases and 12,640 deaths in 2022 [1]. While the incidence of MM has been rising, the number of deaths has not increased in parallel, corresponding to a general improvement in overall survival (OS) as therapeutic advances are made. The development of new effective agents, with 12 MM drugs being approved in the last 15 years and many more in clinical trials, has dramatically changed the therapeutic landscape and has improved patient survivability and outcomes [2]. The novel agents, comprising immunomodulatory drugs (IMiDs) and proteasome inhibitors (PIs), increased in use from 8.7% in 2000 to 61.3% in 2014, and have been shown to improve survival, with patients diagnosed in 2012 being 25% more likely to survive at 2 years than those diagnosed in 2006 [3]. In one study evaluating MM patients diagnosed in 2001–2005 vs. patients diagnosed in 2006–2010, the median OS was 4.6 vs. 6.1 years, respectively, with fewer deaths in the first year after diagnosis in the latter cohort [4]. A separate 2021 analysis from the Multiple Myeloma Research Foundation reported 5-year survival for MM was 53.9%, a significant improvement from the rate of 34.6% in 1998 [2].

Despite the improvement in overall survival rates, the vast majority of patients with myeloma eventually develop disease recurrences, which become increasingly refractory to available anti-MM agents with successive lines of therapy [5]. Relapsed/refractory multiple myeloma (RRMM), defined as disease with continued progression while on therapy or within 60 days of the end of last treatment, poses a significant hurdle for patient survivability with limited effective therapeutic options [6,7,8]. Those patients who have had disease progression during or after treatment with an immunomodulatory agent and proteasome inhibitor are considered to be “double-refractory”; if also resistant to monoclonal antibody treatment, they are deemed “triple-class” refractory and have a particularly grim prognosis. With limited options for next steps, physicians are often unsure on how to proceed, and unfortunately, poor treatment outcomes for this cohort of patients have been the experience, with median OS of around 8 months despite novel therapy use [9]. Without a standard of care for next steps, some physicians elect to reuse previous regimens, especially if the prior response was deep and prolonged; however, this approach has limited data, generally showing short duration of response [10,11]. An alternative strategy for RRMM has been salvage autologous stem cell transplant (ASCT), which has been shown in one study to increase OS in comparison to pulse cyclophosphamide but has not been evaluated in the era of more advanced treatments [12]. One study examining a cohort of patients treated in Australia from 1992–2011 undergoing salvage ASCT who received various induction therapies incorporating vincristine, adriamycin, cyclophosphamide, dexamethasone, as well as thalidomide, found median OS and progression-free survival (PFS) to be 45 and 22 months, respectively [13]. However, a more recent study of salvage ASCT versus immunomodulatory drug-based therapy of lenalidomide/dexamethasone did not show any significant difference in PFS (20.7 months in the transplant arm vs. 18.8 months) or OS (not reached in the transplant arm vs. 62.7 months) [14]. The lack of survival difference seen in this study stems from the failure of nearly 30% of those patients assigned to the salvage ASCT arm not undergoing the treatment, highlighting practical issues with this treatment choice. ASCT is not a feasible treatment option for all patients, with general conditioning, age, and patient preference all contributing to the decision to perform the transplant. Considering continued patient morbidity and mortality as they progress through lines of therapy with diminishing response and survival, more therapeutic strategies are needed to treat RRMM.

Conventional chemotherapy regimens, inhibition of nuclear export, chimeric antigen receptor (CAR)-T and natural killer (NK) cellular therapies, next-generation monoclonal antibodies, and bispecific antibodies have all been studied in recent years and show efficacy in clinical trials in the treatment of triple-refractory MM. What follows is a review and critical evaluation of the most promising of these agents that are likely to be used in future clinical practice.

## 2. Conventional Chemotherapy

Conventional chemotherapy can be used in patients with RRMM as salvage treatment following failure of the novel agents. Dexamethasone, with or without thalidomide, cisplatin, doxorubicin, cyclophosphamide, and etoposide, (D(T)-PACE) or DCEP, has had response rates of approximately 50% in trials [15,16]. However, toxicity is common and PFS is short. Therefore, D(T)-PACE has been most successful when used as a bridge to definitive therapy such as autologous stem cell transplantation [17].

Similar to D(T)-PACE, high-dose cyclophosphamide may also be used as a bridge to other therapy in RRMM. Cyclophosphamide in combination with dexamethasone was shown to be effective in patients with double-refractory MM, with an overall response rate of 55%, with 15% of patients achieving a complete response, and PFS and OS of 6 and 12 months, respectively; however, a separate retrospective analysis concluded that high dose cyclophosphamide use in RRMM should be limited only to patients with good performance status who need immediate therapy and without other reasonable treatment options [18,19].

Bendamustine, a chemotherapy with both alkylating and antimetabolite properties, has also demonstrated efficacy in RRMM [20]. A 2010 retrospective analysis of using bendamustine as the next therapy in heavily pretreated RRMM found 3% of patients achieving a very good partial response, 33% a partial response, 18% a minor response, 26% achieved stable disease, and 20% progressive disease [21]. That study also reported a median event-free survival and OS of 7 and 17 months, respectively; however, these patients had relapsed after a median of only two prior lines of therapy composed of conventional chemotherapy and thalidomide [21]. A more recent retrospective analysis of patients with relapse after a median of four prior lines of therapy, including IMiDs, PIs, and CD38 monoclonal antibodies, showed much inferior outcomes when bendamustine is given as the next line of treatment, with a median PFS and OS of 3.2 and 9.3 months, respectively [22]. Conventional chemotherapy studies discussed in this paper with relevant efficacy and toxicity data are displayed in Table 1.

## 3. Agents with Novel Mechanism of Action

There have been incremental improvements within the classes of immunomodulatory agents and proteasome inhibitors, but cross-resistance occurs and drugs with new mechanisms of action are needed. BCL-2 inhibitors, such as venetoclax, have shown activity in RRMM, especially in those with t (11; 14) mutations with associated high BCL-2 activity [23,24]. In a randomized phase III trial (BELLINI) of venetoclax-bortezomib-dexamethasone vs. bortezomib-dexamethasone, the results showed an improved objective response rate (ORR) of 82% vs. 69% and prolonged PFS of 22.4 months vs. 11.5 months for the venetoclax arm vs. the control arm, respectively [25]. Despite improved efficacy, the FDA placed a partial hold on trials with venetoclax due to increased death from infection with this therapy seen in the BELLINI study. The FDA recommended that all patients with MM receiving venetoclax also receive antibiotic prophylaxis [26]. Further studies of venetoclax in MM are ongoing and currently limited to those patients harboring the t (11; 14) mutation. A phase II study examined the efficacy of venetoclax-dexamethasone treatment in 31 patients having triple-class refractory MM with t (11; 14), with an approximately 50% response rate and PFS of 11 months; however, larger studies are needed to confirm these excellent results [27].

Selinexor is a Selective Inhibitor of Nuclear Export (SINE) that functions by restoring the localization of tumor suppressor proteins, oncogenes, and DNA damage repair complexes through blocking protein transport out of the nucleus into the cytoplasm of the MM cell [28]. In the STORM study, selinexor in combination with low-dose dexamethasone resulted in an ORR of 21% for those with RRMM who had prior treatment with two IMiD and two PI agents (“quad-refractory”) and 20% for quad plus monoclonal antibody (“penta-refractory”) patients. Interestingly, treatment was agnostic of high-risk cytogenetics with 35% ORR for those patients with t (4; 14), t (14; 16), and del (17p) [29]. This study found the median duration of response was 5 months, and 65% of the patients with a treatment response were alive at 12 months [29]. In an extension of the STORM trial, which focused solely on penta-refractory MM, selinexor combined with low-dose dexamethasone yielded an ORR of 26.2% [30]. There is an ongoing phase Ib/II STOMP trial that is studying selinexor and low-dose dexamethasone combined with other MM therapies, including lenalidomide, pomalidomide, bortezomib, carfilzomib, and daratumumab. The study is estimated to be completed in May 2022 (clinical trial number: NCT02343042).

Another novel therapy evaluated for triple-refractory MM is melphalan flufenamide (Melflufen). Melphalan flufenamide is a peptide-drug conjugate that preferentially releases an alkylating moiety into MM cells by exploiting their increased aminopeptidase activity as compared to non-malignant cells [31,32]. The HORIZON and O-12-M1 trials examined melphalan flufenamide as a treatment option for triple-class refractory MM. The phase II HORIZON trial of melphalan flufenamide and dexamethasone in RRMM found an ORR of 29% in the all-treated population and 26% in the triple-refractory population. The median duration of response was 5.5 months, median PFS was 4.2 months, and median OS was 11.6 months [33]. The O-12-M1 trial was a multicenter, international, open-label phase I/II study evaluating melphalan flufenamide plus dexamethasone in the setting of RRMM. In the phase II portion of the study, ORR was 31% and clinical benefit rate was 49% in the all-treated population, while this was 41% and 65%, respectively, in the efficacy-evaluable population [34]. Based on these data, melphalan flufenamide received accelerated approval for triple-class refractory MM on 26 February 2021. This approval was short-lived, however, and melphalan flufenamide was first placed on FDA hold and then taken off the market by the manufacturer after results from the OCEAN (NCT03151811) study showed an increased mortality when melphalan flufenamide was combined with dexamethasone vs. pomalidomide with dexamethasone in triple class refractory MM patients [35].

Yet another novel therapy class under development for RRMM are Cerebos E3 ligase modulators (CELMoD); based on the IMiD platform, CELMoDs are designed for rapid degradation of the proteins Ikaros and Aiolos, which are crucial transcription factors expressed in MM for cell survival and proliferation [36]. The CELMoD CC-92480 was evaluated in RRMM in a multicenter international phase I study and showed a 48% ORR while at a therapeutic dose; at the phase II dose of 1 mg/d orally on Days 1–14 of a 21-day cycle, the response rate (RR) was 55% and disease control rate was 100% [36]. Further studies are ongoing to optimize dose and schedule and partner agents. Another CELMoD named Iberdomide (CC-220) was tested in combination with dexamethasone in a Phase Ib/IIa study and found an ORR of 31%, a minimal response rate or better of 51%, and disease control of 88% [37]. Studies involving novel agents are shown in Table 2, detailing the efficacy and toxicity results. 

## 4. Cellular Therapy

Few treatment breakthroughs in myeloma, let alone oncology in general, have received as much attention recently as CAR-T therapy. Chimeric Antigen Receptor (CAR)-T cells are T cells that have been genetically modified to express an antigen binding region to attach to a target of interest as well as a T cell binding region (CD3), which stimulates T cell cellular cytoxicity [38,39]. CAR-T, thus far, has shown excellent activity in heavily pre-treated MM, and one product, idecabtagene vicleucel (Ide-cel, Abecma^®^) received FDA approval in March 2021 for patients with RRMM who have disease progression after four or more prior lines of therapy, including an IMiD, a PI, and an anti-CD38 monoclonal antibody [39]. The advent of T cell directed therapies in RRMM have brought attention to the uniquely associated toxicities of Cytokine Release Syndrome (CRS) and Immune Effector Cell-Associated Neurotoxicity Syndrome (ICANS). CRS is an inflammatory response driven by persistent reactive cytokine elevation and increased vascular permeability, which can range in severity from mild fever to a systemic shock-like illness [40,41]. Fever was found to be the most common symptom of CRS, with a median onset time of the fever being around day 8.5 after CAR-T infusion for MM patients [40]. The number of plasma cells in the bone marrow may be an independent risk factor for CRS [40]. ICANS may present as headache, confusion, difficulty with word finding and speech, or more severely as encephalopathy and obtundation. ICANS may be difficult to distinguish from CRS and both issues may occur simultaneously or sequentially [42]. The pathophysiology of ICANS is still unclear as well, but it appears in a mouse model to be mediated through inflammatory changes to the endothelium in the blood brain barrier, leading to an increase in the capillary leak of inflammatory cytokines into the central nervous system [43]. Corticosteroids are the mainstay of ICANS treatment, with most patients not experiencing permanent neurological deficits. In RRMM, anti-BCMA CAR T cell therapy has shown overall response rates of 80–100% with a tolerable safety profile [44]. Ide-cel was given to 33 patients with RRMM who had received at least three previous lines of therapy and produced a median PFS of 11.8 months, ORR of 85%, and 45% with CR [41]. After Ide-cel infusion, 76% of patients in this study experienced CRS, and 42% had ICANS, with only 6% and 3% being ≥ Grade 3, in severity, respectively. Another study of Ide-cel found 84% of patients experienced CRS, with 48% maximum grade 1 and 36% maximum grade 2 or higher [45]. Despite the high incidence of CRS and ICANS, these adverse effects were effectively managed using tocilizumab and corticosteroids, and ide-cel therapy was well tolerated for the vast majority of patients tested.

More CAR-T products are far along in clinical development. The phase I/II CARTITUDE-1 study evaluated the activity of Ciltacabtegene autoleucel (Cilta-cel) in triple-class exposed and refractory MM. In 97 patients receiving the cilta-cel infusion, the overall response rate was 97%, with 67% of patients achieving complete response or better. Median progression-free survival has not been reached at time of reporting, and the overall survival rate at 1 year was 89% [46]. CRS was seen in 95% of subjects; however, only 4% was at grade 3 or 4 severity. The CRS after cilta-cel treatment was deemed to be low grade and manageable with a median duration of 4 days, suggesting feasibility of an outpatient dosing regimen which will be further explored in the phase II CARTITUDE-2 study (NCT04133636) [47]. A more recent CAR T cell therapy under development is bb21217, which was modeled after bb2121 but designed to have better cell persistence due to inclusion of a PI3K inhibitor during ex vivo expansion to produce more T cells with a memory phenotype, and has shown a clinical response of 86% in early results of seven patients [48]. P-BCMA-1 is another CAR T cell therapy that has enrichment for memory-like T cells that has shown efficacy with an improved safety profile compared to other CAR T therapy. Emerging data show an ORR of 83%, with only 17% of patients experiencing CRS [49].

Other cell types are under investigation for use as CAR-based therapy, with the primary focus on NK cells both as single agents and in combination with other MM therapy [38]. Preclinical results have shown promise for the potential for CAR-NK therapy targeting CD138, BCMA, NKG2D, and SLAMF7 [50]. CAR-NK candidate NK-92MI showed enhanced cytotoxicity in vitro against CD138^+^ MM cell lines and primary MM cells as compared to empty vector controls [51]. A different candidate CAR-NK cellular therapy, NKTR-255, was shown to improve antitumor efficacy and kinetics when given in combination with CD19 CAR T cells [52]. Another in vitro study of CAR-NK cells targeting SLAMF7 cell therapy demonstrated an enhanced interferon-gamma production and cytotoxic activity against primary MM tumor cells, as well as inhibition of MM tumor growth in a xenograft mouse model [53]. The first CAR-NK clinical trial in MM is currently under way using anti-BCMA CAR-NK (clinical trial number: NCT03940833). Table 3 includes studies on cellular therapy that are discussed in this paper and summarizes the efficacy and toxicity data.

## 5. Immunotherapy

Multiple clinical trials have confirmed the importance of monoclonal antibody treatment in both the upfront and relapsed MM setting [54]. Unfortunately, akin to the experience with conventional chemotherapy, IMiDs, and PIs, treatment resistant disease ultimately develops with monoclonal antibodies, requiring new immunotherapeutic strategies. Programmed cell death protein-1 (PD1) antibodies, such as pembrolizumab, target inhibitory signals to T cells, thereby enhancing T cell mediated cytotoxicity of malignant tumor cells [55]. A reasonable expectation would be that adding another T cell activating agent, such as an IMiD, would increase the anti-tumor effect. A phase II study of pembrolizumab, pomalidomide, and dexamethasone in patients with RRMM found a 60% objective response, with 8% having a complete response, 19% having a very good partial response, and 33% having a partial response [56]. Another study, titled KEYNOTE-023, examined pembrolizumab in combination with lenalidomide and dexamethasone and found an objective response rate of 44% [57]. It should be noted that these studies with pembrolizumab were done in the setting of double-refractory MM, with very few patients having had prior monoclonal antibody exposure. Despite promising efficacy results from early clinical trials, however, a more recent randomized trial revealed unfavorable evidence for the use of PD1 antibodies for RRMM, indicating a PFS of 5.6 months with pembrolizumab + POM/Dex as compared to 8.4 months with POM/Dex alone [58]. It is unlikely that there will be further clinical development of checkpoint inhibitors for MM.

Antibody-drug conjugates combine the specificity of a monoclonal antibody with improved cytotoxicity via targeted delivery of a linked toxin. The first in class antibody-drug conjugate approved for use in RRMM is GSK2857916, or belantamab mafodotin-blmf, also known as the trade name BLENREP^®^, which is an anti-BCMA monoclonal antibody linked to a microtubuledisrupting agent, mono methyl auristatin F (MMAF) [59]. The phase I DREAMM-1 trial investigated belantamab mafodotin as monotherapy and demonstrated a partial response or better in 60% of patients, a median PFS of 12 months, and a median duration of response of 14.3 months [60,61]. The DREAMM-2 trial, a randomized open-label phase II study testing two doses of belantamab mafodotin, also showed efficacy with ORR of 30% for the 2.5 mg/kg cohort and 34% for the 3.4 mg/kg cohort with a manageable safety profile [62]. Of note, all patients in the phase 2 trial, but not the phase I trial, had been previously exposed to or refractory to an anti-CD38 monoclonal antibody. This difference in treatment history for the phase 1 and phase 2 trials could in part explain the discrepancy in efficacy between trials. However, both of these studies were integral in belantamab receiving FDA approval in 2020 for RRMM. Notably, there is a high rate of corneal toxicity, up to 60% of patients in the DREAMM-2 trial, and the requirement of ophthalmologic exams prior to each belantamab dose may make this treatment option impractical in some practice situations [63].

Bispecific antibodies have risen as another exciting novel therapy for triple-class RRMM. Similar to CAR-T, bispecific antibodies function by binding a target on the malignant plasma cells, such as BCMA, GPRC5D, and FcRH5, while simultaneously binding to T cells via CD3 to create an immune synapse leading to immune cell activation and destruction of the cancer cell [64]. There are currently three bispecific antibodies, each with a different target, currently far in clinical development in MM: teclistamab (anti-CD3/BCMA), cevostamab (anti CD3/FcRH5), and talquetamab (anti-CD3/GPRC5D) [64]. G protein coupled-receptor class C group 5 member D (GPRC5D) is a cell surface protein of unknown function that is highly expressed on malignant plasma cells. In a study that was presented at the ASCO 2020 annual Meeting, talquetamab had an ORR in response-evaluable patients of 63%, with 50% with very good partial response or better, and a tolerable safety profile without any dose-limiting toxicities [65]. Teclistamab, a BCMA/CD3 bispecific antibody, is another drug in this class that had 63.8% ORR, with 51% with a very good partial response or better and 19% with a complete response or better and also with a tolerable safety profile [66]. The target of cevostamab is Fc Receptor Homolog 5 (FcRH5), a protein that is expressed exclusively in B-lineage cells and universally expressed in MM cells [67]. FcRH5 has a role in isotype selection and proliferation in activated B cells [68]. FcRH5 is encoded on chromosome 1q21 and 1q21 amplifications are a negative prognostic factor in MM, suggesting a role for FcRH5 in MM survival [69]. In the 2020 ASH report of a phase I study of cevostamab in RRMM patients with a median of six prior lines of therapy, the ORR was 51.7% and median duration of response has not been reached, with some patients responding for longer than 1 year [70].

Bispecific T Cell engagers (BiTE) technology are peptide molecules with two variable region binding domains, similar to bispecific monoclonal antibodies targeting a T cell and a tumor antigen simultaneously, meant to induce T cell mediated cytotoxicity against the cancer cell [71]. In RRMM, preliminary results from the first human study showed that AMG 420, an anti-BCMA bispecific T cell engager, showed promising activity, with 31% of patients achieving a response and the median time to any response of 1 month [72]. Despite the encouraging preliminary results on response, the study also reported serious adverse events in 50% of the patients, with infection being the leading cause [72]. Later trial results of AMG 420 revealed a response rate of 70% at the maximum tolerated dose, with 50% having minimal residual disease (MRD)-negative complete response [73]. Despite the efficacy, AMG 420 has been discontinued from development because of its short half-life leading to a continuous infusion pump requirement and, thus, to being burdensome for patients and clinical practices. A follow-up molecule, AMG 701, was developed with the inclusion of an antibody Fc region, which had a longer half-life, to allow for more convenient patient dosing. A phase I study of AMG 701 demonstrated an initial response rate of 36% overall and a response rate of 83% after dose escalation, with a median time to response of 1 month and time to best response of 2.8 months [74]. Further trials of AMG 701 are under way.

Similar to the experience with CAR-T, both bispecific antibodies and BITE molecules may be associated with CRS, although to a lesser degree and duration. AMG701 had an overall rate of CRS of 65%, with only 9% of moderate severity or greater; teclistamab and talquetamab were associated with an approximate 55% rate of CRS, with very few cases that were more than mild in intensity [66]. The anti-FcRH5 bispecific cevostamab had a higher reported rate of CRS at 76%, also with few severe cases [70]. For the BITE and bispecific treatments, the onset of CRS was quicker with intravenous vs. subcutaneous administration (24 vs. 48 h) and median duration of symptoms was 12–24 h. CRS tended not to recur with dosing beyond the first instance. In the BITE and bispecific studies, the ICANS rate was low with teclistamab and talquetamab at 5% and 6%, respectively [66]. Cevostamab was associated with a higher rate of neurotoxicity at 28%, with very few severe cases [70]. CAR-T is a single infusion treatment that requires patient planning and manufacturing time; bispecifics and BITEs are “off the shelf” therapies that can be given quickly to patients in need. All three immunotherapy modalities are complementary to each other and will be important additions to the MM armamentarium. Table 4 includes studies pertaining to immunotherapy discussed in this paper, outlining efficacy and toxicity data.

## 6. Discussion

Despite the increasing number of therapies available to treat myeloma, triple-refractory MM still poses a significant challenge to treat due to limited options available for next steps and due to the lack of guidelines available to direct management. Conventional chemotherapy is one option available to these patients, but data in previously untreated patients have shown that it does not significantly improve survival when compared to melphalan and prednisone, and increasing the dosing on chemotherapy has not resulted in significant differences in response duration and survival; additionally, there is limited evidence of efficacy in the context of RRMM, and therefore, it is of unknown benefit to patients. [75]. Salvage ASCT is also an approach for triple-refractory MM, but the response duration is limited and not all patients will be eligible.

Novel agents are needed to provide better treatment options for patients with triple-refractory myeloma. There are ongoing clinical trials for novel-pathway agents, such as selinexor, monoclonal antibodies and next-generation antibodies, bispecific antibodies, as well as cellular therapy using CAR-T and CAR-NK cells. CAR-T cell therapy is a breakthrough option that many deem to be the future for myeloma treatment with eventual movement into earlier lines of therapy [38]. However, the manufacturing process is laborious, taking 2–4 weeks for completion, with the waiting list of patients growing longer as manufacturer supply issues linger. CAR NK-cell therapy is another potential future direction for MM therapy with promising preclinical results, but the early clinical trials are ongoing, with more mature results yet to be seen. Even after these clinical trials result, however, the same laborious process most likely will apply to the production of CAR NK-cell therapy, requiring improved measures for making this therapy available to patients with MM.

In addition to the slow manufacturing and limited process for manufacture of CAR T cells, another challenge for the development of newer therapies is the toxicity and need for greater supportive care. For example, venetoclax was placed on hold for the treatment of MM due to the greater risk of dying from infection, requiring antibiotics to be administered in adjunct with this therapy. In addition to the risk of infection due to both immunosuppression and myelosuppression, CRS is another dangerous toxicity in therapies for MM, such as CAR T cells, BiTEs, and bispecific antibodies. Although fortunately seen in <5% of patients, CRS may be severe and can progress to an uncontrolled systemic inflammatory response with circulatory shock that requires vasopressors, vascular leakage, disseminated intravascular coagulation, multi-organ failure, and possibly death [76]. First-line treatment of CRS with tocilizumab, through its ability to block IL-6 released from macrophages during CRS, has made this toxicity manageable [77]. Although CRS is able to be controlled with therapy and most patients do not have lasting clinical consequences, there is no consensus regarding monitoring CRS or determining when CRS is resolved, necessitating guidelines especially as the use of CAR-T cell therapy increasingly becomes a mainstay of practice.

CAR-T and the other therapies can produce meaningful responses; however, the durations of response are limited. More research is still needed in the setting of triple-refractory MM to discover other approaches to treatment.

In addition to the promising studies on CAR-T cell technology, antibody-drug conjugates and bispecific antibodies have also demonstrated excellent efficacy, with good tolerability in RRMM. The sequencing of these agents in relationship to CAR-T remains to be determined and will likely be influenced by patient comorbidities, disease characteristics, and local availability of the services needed to deliver cellular therapies. There is also the unknown relationship between prior BCMA targeted therapy, MoAb history, and T cell directed agents; whether prior exposure to any of these modalities would affect future efficacy remains to be determined. While new data are being garnered, the decision of which agents to use is based on practical use needs.

Cytogenic abnormalities or other disease-related factors, treatment-related factors, such as prior drug exposures and toxicities, longevity of prior remission, and retreatment, and patient-related factors, such as renal insufficiency, hepatic impairment, comorbidities, and patient preference, should all be considered in deciding therapy for patients with triple-refractory MM [78]. Further studies will focus on identification of new biomarkers for novel therapies in the advancement of finding a cure [79].

## 7. Conclusions

Treatment paradigms for relapsed or refractory myeloma have changed rapidly from the use of chemotherapeutics, to novel agents, and now immune-based therapies. Myeloma that is refractory to an immunomodulatory agent, a proteasome inhibitor, and monoclonal anti-CD38 antibody treatment (triple-class refractory) myeloma has emerged as a pressing need to find new effective management strategies. The next generation of novel agents with new mechanisms of action, such as venetoclax and selinexor, has shown some activity in triple class refractory myeloma but optimal use is still under testing. Cellular treatments with CAR-T as well as T cell-engaging therapies have proven to be highly active in heavily pretreated relapsed myeloma. More data regarding long-term efficacy and adverse event management with the use of immune-based therapies is currently being generated. Next-generation cellular and engineered monoclonal antibody therapies are also under testing and development.

## Figures and Tables

**Table 1:** Conventional chemotherapy regimen clinical trial results in relapsed or refractory multiple myeloma.

Conventional Chemotherapy	Study	Median Prior Lines of Therapy	ORR (%)	PFS (Months, %)	OS (Months, %)	Most Common Grade ≥ 3 Toxicity (%)
**Dexamethasone, thalidomide, cisplatin, doxorubicin, cyclophosphamide, and etoposide [D(T)PACE]**	Gerrie et al., 2013 [15]	3	49%	5.5 months	14.0 months	neutropenia (84%), thrombocytopenia (70%), anemia (48%)
**Dexamethasone, cisplatin, doxorubicin, cyclophosphamide, and etoposide** **(DPACE)**	Ronchetti et al., 2013 [16]	2	50%	5.2 months	6.7 months	neutropenia (100%), thrombocytopenia (82%), anemia (64%)
**High-dose cyclophosphamide with dexamethasone**	Nikonova et al., 2016 [18]	4	55%	6.0 months	12.0 months	neutropenia (78%), febrile neutropenia (72%)
**High-dose cyclophosphamide, bortezomib, doxorubicin, and dexamethasone (mCBAD)**	Tabchi et al., 2019 [19]	3	85%	4.64 months	13.96 months	anemia (97%), neutropenia (95%), thrombocytopenia (94%)
**Bendamustine**	Michael et al., 2010 [21]	2	36%	7.0 months	17.0 months	neutropenia (41%), thrombocytopenia (26%), infection (15%), anemia (10%)
**Bendamustine**	Gandhi et al., 2019 [22]	4	33.3%	3.2 months	9.3 months	Not reported

**Table 2:** Novel agent regimen clinical trial results in relapsed or refractory multiple myeloma.

Novel Agents	Study	Other Regimen Drugs	Median Prior Lines of Therapy	ORR (%)	PFS (Months, %)	OS (Months, %)	Most Common Grade ≥ 3 Toxicity (%)
**Venetoclax**	BELLINI Phase III [25]	Bortezomib, DEX	1–3	82%	22.4 months	Not reached	neutropenia (18%), pneumonia (16%) thrombocytopenia (15%), anemia (15%), diarrhea (15%)
**Venetoclax**	Kaufman et al., 2021 Phase II [27]	DEX	3	48%	10.8 months	77%	lymphopenia (19%), neutropenia (7%), thrombocytopenia (10%), anemia (16%)
**Selinexor**	STORM Phase II [29]	DEX	5	21%	2.3 months	9.3 months	thrombocytopenia (59%), anemia (28%), neutropenia (23%), hyponatremia (22%), leukopenia (15%)
**Melphalan flufenamide**	HORIZON Phase II [33]	DEX	5	26%	4.2 months	11.6 months	neutropenia (79%), thrombocytopenia (76%), anemia (43%), pneumonia (10%)
**Melphalan flufenamide**	O-12-M1 Phase I/II [34]	DEX	4	41%	Ongoing	ongoing	neutropenia (58%), thrombocytopenia (62%), pneumonia (11%)
**Melphalan flufenamide**	OCEAN Phase III [35]	DEX	3	32%	6.8 months	19.8 months	thrombocytopenia (63%), neutropenia (54%), anemia (43%)
**CC-92480**	Richardson et al., 2020 Phase I/II [36]	DEX	6	48%	Ongoing	Ongoing	neutropenia (53%), infections (30%), anemia (29%), thrombocytopenia (17%)
**Iberdomide (CC-220)**	Lonial et al., 2019 Phase I/II [37]	DEX	5	31%	Ongoing	Ongoing	neutropenia (26%), thrombocytopenia (11%), neuropathy (2%)

DEX: dexamethasone.

**Table 3:** Cellular therapy clinical trial results in relapsed or refractory multiple myeloma.

Cellular Therapy	Study	Median Prior Lines of Therapy	ORR (%)	PFS (Months, %)	OS (Months, %)	Most Common Grade ≥ 3 Toxicity (%)
**Ide-Cel (bb2121)**	Raje et al., 2019 [41]	7	85%	11.8 months	Not reached	neutropenia (85%), leukopenia (58%), anemia (45%), thrombocytopenia (45%), CRS (6%), ICANS (3%)
**Cilta-Cel**	CARTITUDE-I Phase I/II [46]	6	97%	77%	89%	neutropenia (95%), anemia (68%), leukopenia (61%), thrombocytopenia (60%), lymphopenia (50%), CRS (4%), ICANS (9%).
**bb21217**	Shah et al., 2018 [48]	9	86%	Ongoing	Ongoing	CRS (14%)
**P-BCMA-101**	Gregory et al., 2018 Phase I [49]	3–9	83%	Ongoing	Ongoing	cytopenias, febrile neutropenia (%not reported)

**Table 4:** T cell-directed immunotherapy clinical trial results in relapsed or refractory multiple myeloma.

T Cell-Directed Immunotherapy	Study	Other Regimen Drugs	Median Prior Lines of Therapy	ORR (%)	PFS (Months, %)	OS (Months, %)	Most Common Grade ≥ 3 Toxicity (%)
**Pembrolizumab**	Badros et al., 2017 Phase II [56]	POM/DEX	3	60%	17.4 months	Not reached	Hematologic toxicity (40%), hyperglycemia (25%), pneumonia (15%)
**Pembrolizumab**	KEYNOTE-023 Phase I [57]	LEN/DEX	2–5+	44%	7.2 months	Not reached	Neutropenia (27.4%), thrombocytopenia (16.1%), anemia (8.1%)
**Pembrolizumab**	KEYNOTE-183 Phase III [58]	POM/DEX	2–4	34%	5.6 months	82% estimated	Neutropenia (34%), anemia (17%), pneumonia (13%), pneumonia (13%), thrombocytopenia (12%)
**Belantamab**	DREAMM-1 Phase I [60,61]	none	5	60%	12 months	Not reported	Thrombocytopenia (34%), anemia (17%), pneumonia (9%)
**Belantamab**	DREAMM-2 Phase II [62]	none	7	34%	Ongoing	Ongoing	Keratopathy (27%), thrombocytopenia (33%), anemia (25%)
**Talquetamab**	Berdeja et al., 2021 Phase I [65]	none	4	63%	Ongoing	Ongoing	CRS (4%), neutropenia (54%), anemia (29%)
**Teclistamab**	Garfall et al., 2020 Phase I [66]	none	6	63.8%	Ongoing	Ongoing	Neutropenia (23%), anemia (9%)
**Cevostamab**	Cohen et al., 2020 Phase I [70]	none	6	51.7%	Ongoing	Ongoing	Lymphopenia (11.8%), neutropenia (9.8%), anemia (5.9%), thrombocytopenia (5.9%)
**AMG-420**	Topp et al., 2020 Phase I [72]	none	4	70%	Ongoing	ongoing	Infections (33%), polyneuropathy (5%)
**AMG-701**	Harrison et al., 2020 Phase I [74]	none	6	83%	Ongoing	Ongoing	Anemia (43%), neutropenia (23%), thrombocytopenia (20%), CRS (7%)

DEX: dexamethasone; LEN: lenalidomide; POM: pomalidomide; CRS: Cytokine Release Syndrome.

## Data Availability

Not applicable.

## References

[1] Key Statistics About Multiple Myeloma. https://www.cancer.org/cancer/multiple-myeloma/about/key-statistics.html..

[2] Relative Survival for Multiple Myeloma. https://themmrf.org/multiple-myeloma/prognosis/understanding-survival-statistics/.

[3] Fonseca R., Abouzaid S., Bonafede M., Cai Q., Parikh K., Cosler L., Richardson P. (2017). Trends in overall survival and costs of multiple myeloma, 2000–2014. Leukemia.

[4] Kumar S.K., Dispenzieri A., Lacy M.Q., Gertz M.A., Buadi F.K., Pandey S.C., Kapoor P., Dingli D., Hayman S.R., Leung N. (2013). Continued improvement in survival in multiple myeloma: Changes in early mortality and outcomes in older patients. Leukemia.

[5] Kyle R.A., Rajkumar S.V. (2008). Multiple myeloma. Blood.

[6] Anderson K.C., Kyle R.A., Rajkumar S.V., Stewart A.K., Weber D., Richardson P. (2007). Myeloma, Clinically relevant end points and new drug approvals for myeloma. Leukemia.

[7] Kumar S.K., Lee J.H., Lahuerta J.J., Morgan G., Richardson P.G., Crowley J., Haessler J., Feather J., Hoering A., Moreau P. (2012). Risk of progression and survival in multiple myeloma relapsing after therapy with IMiDs and bortezomib: A multicenter international myeloma working group study. Leukemia.

[8] Kumar S.K., Dimopoulos M.A., Kastritis E., Terpos E., Nahi H., Goldschmidt H., Hillengass J., Leleu X., Beksac M., Alsina M. (2017). Natural history of relapsed myeloma, refractory to immunomodulatory drugs and proteasome inhibitors: A multicenter IMWG study. Leukemia.

[9] Usmani S., Ahmadi T., Ng Y., Lam A., Desai A., Potluri R., Mehra M. (2016). Analysis of Real-World Data on Overall Survival in Multiple Myeloma Patients With ≥3 Prior Lines of Therapy Including a Proteasome Inhibitor (PI) and an Immunomodulatory Drug (IMiD), or Double Refractory to a PI and an IMiD. Oncologist.

[10] Petrucci M.T., Giraldo P., Corradini P., Teixeira A., Dimopoulos M., Blau I.W., Drach J., Angermund R., Allietta N., Broer E. (2013). A prospective, international phase 2 study of bortezomib retreatment in patients with relapsed multiple myeloma. Br. J. Haematol..

[11] Mikhael J.R. (2014). A practical approach to relapsed multiple myeloma. Hematology.

[12] Cook G., Ashcroft A.J., Cairns D.A., Williams C.D., Brown J.M., Cavenagh J.D., Snowden J.A., Parrish C., Yong K., Cavet J. (2016). The effect of salvage autologous stem-cell transplantation on overall survival in patients with relapsed multiple myeloma (final results from BSBMT/UKMF Myeloma X Relapse [Intensive]): A randomised, open-label, phase 3 trial. Lancet Haematol..

[13] Chow A.W.S., Lee C.H.S., Hiwase D.K., To L.B., Horvath N. (2012). Relapsed multiple myeloma: Who benefits from salvage autografts?. Intern. Med. J..

[14] Goldschmidt H., Baertsch M.-A., Schlenzka J., Becker N., Habermehl C., Hielscher T., Raab M.-S., Hillengass J., Sauer S., Müller-Tidow C. (2021). Salvage autologous transplant and lenalidomide maintenance vs. lenalidomide/dexamethasone for relapsed multiple myeloma: The randomized GMMG phase III trial ReLApsE. Leukemia.

[15] Gerrie A.S., Mikhael J.R., Cheng L., Jiang H., Kukreti V., Panzarella T., Reece D., Stewart K.A., Trieu Y., Trudel S. (2013). D(T)PACE as salvage therapy for aggressive or refractory multiple myeloma. Br. J. Haematol..

[16] Ronchetti A.-M., Isnard F., Buffet M., Coman T., Gorin N.-C., Coppo P., Garderet L., Malak S. (2012). Dexamethasone, cisplatin, doxorubicin, cyclophosphamide and etoposide (DPACE) is an effective salvage regimen for multiple myeloma refractory to novel agents. Leuk. Lymphoma.

[17] Toocheck C., Pinkhas D. (2018). Treatment of relapsed multiple myeloma complicated by cardiac extramedullary plasmacytoma with D-PACE chemotherapy. BMJ Case Rep..

[18] Nikonova A., Caplan S.N., Shamy A., Gyger M. (2016). High-Dose Cyclophosphamide in Highly Refractory Multiple Myeloma Patients as a Bridge to Further Novel Therapies. Blood.

[19] Tabchi S., Nair R., Kunacheewa C., Patel K.K., Lee H.C., Thomas S.K., Amini B., Ahmed S., Mehta R.S., Bashir Q. (2019). Retrospective Review of the Use of High-Dose Cyclophosphamide, Bortezomib, Doxorubicin, and Dexamethasone for the Treatment of Multiple Myeloma and Plasma Cell Leukemia. Clin. Lymphoma Myeloma Leuk..

[20] Cheson B.D., Rummel M.J. (2009). Bendamustine: Rebirth of an Old Drug. J. Clin. Oncol..

[21] Michael M., Bruns I., Bölke E., Zohren F., Czibere A., Safaian N.N., Neumann F., Haas R., Kobbe G., Fenk R. (2010). Bendamustine in patients with relapsed or refractory multiple myeloma. Eur. J. Med Res..

[22] Gandhi U.H., Cornell R.F., Lakshman A., Gahvari Z.J., McGehee E., Jagosky M.H., Gupta R., Varnado W., Fiala M.A., Chhabra S. (2019). Outcomes of patients with multiple myeloma refractory to CD38-targeted monoclonal antibody therapy. Leukemia.

[23] Kumar S., Vij R., Kaufman J.L., Mikhael J., Facon T., Pegourie B., Benboubker L., Gasparetto C., Amiot M., Moreau P. (2016). Venetoclax Monotherapy for Relapsed/Refractory Multiple Myeloma: Safety and Efficacy Results from a Phase I Study. Blood.

[24] Kumar S., Kaufman J.L., Gasparetto C., Mikhael J., Vij R., Pegourie B., Benboubker L., Facon T., Amiot M., Moreau P. (2017). Efficacy of venetoclax as targeted therapy for relapsed/refractory t(11;14) multiple myeloma. Blood.

[25] Kumar S.K., Harrison S.J., Cavo M., de la Rubia J., Popat R., Gasparetto C., Hungria V., Salwender H., Suzuki K., Kim I. (2020). Venetoclax or placebo in combination with bortezomib and dexamethasone in patients with relapsed or refractory multiple myeloma (BELLINI): A randomised, double-blind, multicentre, phase 3 trial. Lancet Oncol..

[26] FDA Warns about the Risks Associated with the Investigational Use of Venclexta in Multiple Myeloma. https://www.fda.gov/Drugs/DrugSafety/ucm634120.htm.

[27] Kaufman J.L., Gasparetto C., Schjesvold F.H., Moreau P., Touzeau C., Facon T., Boise L.H., Jiang Y., Yang X., Dunbar F. (2020). Targeting BCL -2 with venetoclax and dexamethasone in patients with relapsed/refractory t(11;14) multiple myeloma. Am. J. Hematol..

[28] Richter J., Madduri D., Richard S., Chari A. (2020). Selinexor in relapsed/refractory multiple myeloma. Ther. Adv. Hematol..

[29] Vogl D.T., Dingli D., Cornell R.F., Huff C.A., Jagannath S., Bhutani D., Zonder J., Baz R., Nooka A., Richter J. (2018). Selective Inhibition of Nuclear Export with Oral Selinexor for Treatment of Relapsed or Refractory Multiple Myeloma. J. Clin. Oncol..

[30] Chari A., Vogl D.T., Dimopoulos M.A., Nooka A.K., Huff C.A., Moreau P., Cole C.E., Richter J., Dingli D., Vij R. (2018). Results of the Pivotal STORM Study (Part 2) in Penta-Refractory Multiple Myeloma (MM): Deep and Durable Responses with Oral Selinexor Plus Low Dose Dexamethasone in Patients with Penta-Refractory MM. Blood.

[31] Miettinen J., Kumari R., Traustadottir G., Huppunen M.-E., Sergeev P., Majumder M., Schepsky A., Gudjonsson T., Lievonen J., Bazou D. (2021). Aminopeptidase Expression in Multiple Myeloma Associates with Disease Progression and Sensitivity to Melflufen. Cancers.

[32] Mateos M.-V., Bladé J., Bringhen S., Ocio E.M., Efebera Y., Pour L., Gay F., Sonneveld P., Gullbo J., Richardson P.G. (2020). Melflufen: A Peptide–Drug Conjugate for the Treatment of Multiple Myeloma. J. Clin. Med..

[33] Richardson P.G., Oriol A., Larocca A., Bladé J., Cavo M., Rodriguez-Otero P., Leleu X., Nadeem O., Hiemenz J.W., Hassoun H. (2021). Melflufen and Dexamethasone in Heavily Pretreated Relapsed and Refractory Multiple Myeloma. J. Clin. Oncol..

[34] Richardson P.G., Bringhen S., Voorhees P., Plesner T., Mellqvist U.-H., Reeves B., Paba-Prada C., Zubair H., Byrne C., Chauhan D. (2020). Melflufen plus dexamethasone in relapsed and refractory multiple myeloma (O-12-M1): A multicentre, international, open-label, phase 1–2 study. Lancet Haematol..

[35] Schjesvold F., Dimopoulos M.-A., Delimpasi S., Robak P., Coriu D., Legiec W., Pour L., Špička I., Masszi T., Doronin V. (2021). OAB-050: OCEAN (OP-103): A Phase 3, randomized, global, head-to-head comparison study of Melflufen and Dexamethasone (Dex) versus Pomalidomide (Pom) and Dex in Relapsed Refractory Multiple Myeloma (RRMM). Clin. Lymphoma Myeloma Leuk..

[36] Richardson P.G., Vangsted A.J., Ramasamy K., Trudel S., Martínez J., Mateos M.-V., Rodríguez Otero P., Lonial S., Popat R., Oriol A. (2020). First-in-human phase I study of the novel CELMoD agent CC-92480 combined with dexamethasone (DEX) in patients (pts) with relapsed/refractory multiple myeloma (RRMM). J. Clin. Oncol..

[37] Lonial S., Van De Donk N.W., Popat R., Zonder J.A., Minnema M.C., Larsen J., Nguyen T.V., Chen M.S., Bensmaine A., Cota M. (2019). First clinical (phase 1b/2a) study of iberdomide (CC-220; IBER), a CELMoD, in combination with dexamethasone (DEX) in patients (pts) with relapsed/refractory multiple myeloma (RRMM). J. Clin. Oncol..

[38] Teoh P.J., Chng W.J. (2021). CAR T-cell therapy in multiple myeloma: More room for improvement. Blood Cancer J..

[39] Mikkilineni L., Kochenderfer J.N. (2017). Chimeric antigen receptor T-cell therapies for multiple myeloma. Blood.

[40] Yan Z., Zhang H., Cao J., Zhang C., Liu H., Huang H., Cheng H., Qiao J., Wang Y., Wang Y. (2021). Characteristics and Risk Factors of Cytokine Release Syndrome in Chimeric Antigen Receptor T Cell Treatment. Front. Immunol..

[41] Raje N., Berdeja J., Lin Y., Siegel D., Jagannath S., Madduri D., Liedtke M., Rosenblatt J., Maus M.V., Turka A. (2019). Anti-BCMA CAR T-Cell Therapy bb2121 in Relapsed or Refractory Multiple Myeloma. N. Engl. J. Med..

[42] Morris E.C., Neelapu S.S., Giavridis T., Sadelain M. (2021). Cytokine release syndrome and associated neurotoxicity in cancer immunotherapy. Nat. Rev. Immunol..

[43] Gust J., Hay K.A., Hanafi L.-A., Li D., Myerson D., Gonzalez-Cuyar L.F., Yeung C., Liles W.C., Wurfel M., Lopez J.A. (2017). Endothelial Activation and Blood–Brain Barrier Disruption in Neurotoxicity after Adoptive Immunotherapy with CD19 CAR-T Cells. Cancer Discov..

[44] Cho S.-F., Anderson K.C., Tai Y.-T. (2018). Targeting B Cell Maturation Antigen (BCMA) in Multiple Myeloma: Potential Uses of BCMA-Based Immunotherapy. Front. Immunol..

[45] Kansagra A., Lin Y., Berdeja J.G., Shah N., Oriol A., Yakoub-Agha I., Einsele H., Rambaldi A., Truppel-Hartmann A., Rowe E. (2020). Characterization of Cytokine Release Syndrome in the KarMMa Study of Idecabtagene Vicleucel (ide-cel, bb2121) for Relapsed and Refractory Multiple Myeloma. Blood.

[46] Berdeja J.G., Madduri D., Usmani S.Z., Jakubowiak A., Agha M., Cohen A.D., Stewart A.K., Hari P., Htut M., Lesokhin A. (2021). Ciltacabtagene autoleucel, a B-cell maturation antigen-directed chimeric antigen receptor T-cell therapy in patients with relapsed or refractory multiple myeloma (CARTITUDE-1): A phase 1b/2 open-label study. Lancet.

[47] Lin Y., Martin I.T., Cohen A.D., Jakubowiak A., Jasielec J., Usmani M.S.Z., Madduri D., Agha M., Stewart M.A.K., Singh I. (2020). Cytokine Release Syndrome in Patients with Relapsed/Refractory Multiple Myeloma Treated with Ciltacabtagene Autoleucel in the Phase 1b/2 CARTITUDE-1 Study. Blood.

[48] Shah N., Alsina M., Siegel D.S., Jagannath S., Madduri D., Kaufman J.L., Turka A., Lam L.P., Massaro M.M., Hege K. (2018). Initial Results from a Phase 1 Clinical Study of bb21217, a Next-Generation Anti Bcma CAR T Therapy. Blood.

[49] Gregory T., Cohen A.D., Costello C.L., Ali S.A., Berdeja J.G., Ostertag E.M., Martin C., Shedlock D.J., Resler B.M.L., Spear M.A. (2018). Efficacy and Safety of P-Bcma-101 CAR-T Cells in Patients with Relapsed/Refractory (r/r) Multiple Myeloma (MM). Blood.

[50] Alfarra H., Weir J., Grieve S., Reiman T. (2020). Targeting NK Cell Inhibitory Receptors for Precision Multiple Myeloma Immunotherapy. Front. Immunol..

[51] Jiang H., Zhang W., Shang P., Zhang H., Fu W., Ye F., Zeng T., Huang H., Zhang X., Sun W. (2013). Transfection of chimeric anti-CD138 gene enhances natural killer cell activation and killing of multiple myeloma cells. Mol. Oncol..

[52] Chou C., Fräßle S.P., Hawkins R.M., Steinmetz R., Phi T.-D., Busch D.H., Miyazaki T., Marcondes M., Riddell S.R., Turtle C.J. (2019). Effects of NKTR-255, a polymer conjugated human IL-15, on efficacy of CD19 CAR T CELL immunotherapy in a preclinical Lymphoma model. HemaSphere.

[53] Chu J., Deng Y., Benson D.M., He S., Hughes T.P., Zhang J., Peng Y., Mao H., Yi L., Ghoshal K. (2013). CS1-specific chimeric antigen receptor (CAR)-engineered natural killer cells enhance in vitro and in vivo antitumor activity against human multiple myeloma. Leukemia.

[54] Sherbenou D., Mark T.M., Forsberg P. (2017). Monoclonal Antibodies in Multiple Myeloma: A New Wave of the Future. Clin. Lymphoma Myeloma Leuk..

[55] Rosenblatt J., Avigan D. (2017). Targeting the PD-1/PD-L1 axis in multiple myeloma: A dream or a reality?. Blood.

[56] Badros A., Hyjek E., Ma N., Lesokhin A., Dogan A., Rapoport A.P., Kocoglu M., Lederer E., Philip S., Milliron T. (2017). Pembrolizumab, pomalidomide, and low-dose dexamethasone for relapsed/refractory multiple myeloma. Blood.

[57] Mateos M.-V., Orlowski R.Z., Ocio E.M., Rodríguez-Otero P., Reece D., Moreau P., Munshi N., Avigan D.E., Siegel D.S., Ghori R. (2019). Pembrolizumab combined with lenalidomide and low-dose dexamethasone for relapsed or refractory multiple myeloma: Phase I KEYNOTE -023 study. Br. J. Haematol..

[58] Mateos M.-V., Blacklock H., Schjesvold F., Oriol A., Simpson D., George A., Goldschmidt H., LaRocca A., Chanan-Khan A., Sherbenou D. (2019). Pembrolizumab plus pomalidomide and dexamethasone for patients with relapsed or refractory multiple myeloma (KEYNOTE-183): A randomised, open-label, phase 3 trial. Lancet Haematol..

[59] Tai Y.-T., Mayes P.A., Acharya C., Zhong M.Y., Cea M., Cagnetta A., Craigen J., Yates J., Gliddon L., Fieles W. (2014). Novel anti–B-cell maturation antigen antibody-drug conjugate (GSK2857916) selectively induces killing of multiple myeloma. Blood.

[60] Trudel S., Lendvai N., Popat R., Voorhees P.M., Reeves B., Libby E.N., Richardson P.G., Anderson L., Sutherland H.J., Yong K. (2018). Targeting B-cell maturation antigen with GSK2857916 antibody–drug conjugate in relapsed or refractory multiple myeloma (BMA117159): A dose escalation and expansion phase 1 trial. Lancet Oncol..

[61] Trudel S., Lendvai N., Popat R., Voorhees P.M., Reeves B., Libby E.N., Richardson P.G., Hoos A., Gupta I., Bragulat V. (2019). Antibody–drug conjugate, GSK2857916, in relapsed/refractory multiple myeloma: An update on safety and efficacy from dose expansion phase I study. Blood Cancer J..

[62] Lonial S., Lee H.C., Badros A., Trudel S., Nooka A.K., Chari A., Abdallah A.-O., Callander N., Lendvai N., Sborov D. (2019). Belantamab mafodotin for relapsed or refractory multiple myeloma (DREAMM-2): A two-arm, randomised, open-label, phase 2 study. Lancet Oncol..

[63] Ms S.V.P., Joshi N., Thareja T., Jhanji V. (2021). Corneal epithelial toxicity induced by belantamab mafodotin. Clin. Exp. Ophthalmol..

[64] Lancman G., Sastow D.L., Cho H.J., Jagannath S., Madduri D., Parekh S.S., Richard S., Richter J., Sanchez L., Chari A. (2021). Bispecific Antibodies in Multiple Myeloma: Present and Future. Blood Cancer Discov..

[65] Berdeja J.G., Krishnan A.Y., Oriol A., van de Donk N.W.C.J., Rodríguez-Otero P., Askari E., Mateos M.-V., Minnema M.C., Costa L.J., Verona R. (2021). Updated results of a phase 1, first-in-human study of talquetamab, a G protein-coupled receptor family C group 5 member D (GPRC5D) × CD3 bispecific antibody, in relapsed/refractory multiple myeloma (MM). J. Clin. Oncol..

[66] Garfall A.L., Usmani S.Z., Mateos M.-V., Nahi H., Van De Donk N.W., San-Miguel J.F., Rocafiguera A.O., Rosinol L., Chari A., Bhutani M. (2020). Updated Phase 1 Results of Teclistamab, a B-Cell Maturation Antigen (BCMA) x CD3 Bispecific Antibody, in Relapsed and/or Refractory Multiple Myeloma (RRMM). Blood.

[67] Elkins K., Zheng B., Go M., Slaga D., Du C., Scales S.J., Yu S.-F., McBride J., de Tute R., Rawstron A. (2012). FcRL5 as a Target of Antibody–Drug Conjugates for the Treatment of Multiple Myeloma. Mol. Cancer Ther..

[68] Polson A.G., Zheng B., Elkins K., Chang W., Du C., Dowd P., Yen L., Tan C., Hongo J.-A., Koeppen H. (2006). Expression pattern of the human FcRH/IRTA receptors in normal tissue and in B-chronic lymphocytic leukemia. Int. Immunol..

[69] Inoue J., Otsuki T., Hirasawa A., Imoto I., Matsuo Y., Shimizu S., Taniwaki M., Inazawa J. (2004). Overexpression of PDZK1 within the 1q12-q22 Amplicon Is Likely To Be Associated with Drug-Resistance Phenotype in Multiple Myeloma. Am. J. Pathol..

[70] Cohen A.D., Harrison S.J., Krishnan A., Fonseca R., Forsberg P.A., Spencer A., Berdeja J.G., Laubach J.P., Li M., Choeurng V. (2020). Initial Clinical Activity and Safety of BFCR4350A, a FcRH5/CD3 T-Cell-Engaging Bispecific Antibody, in Relapsed/Refractory Multiple Myeloma. Blood.

[71] Nishida H. (2021). Rapid Progress in Immunotherapies for Multiple Myeloma: An Updated Comprehensive Review. Cancers.

[72] Topp M.S., Duell J., Zugmaier G., Attal M., Moreau P., Langer C., Kroenke J., Facon T., Salnikov A., Lesley R. (2019). Evaluation of AMG 420, an anti-BCMA bispecific T-cell engager (BiTE) immunotherapy, in R/R multiple myeloma (MM) patients: Updated results of a first-in-human (FIH) phase I dose escalation study. J. Clin. Oncol..

[73] Topp M.S., Duell J., Zugmaier G., Attal M., Moreau P., Langer C., Krönke J., Facon T., Salnikov A.V., Lesley R. (2020). Anti–B-Cell Maturation Antigen BiTE Molecule AMG 420 Induces Responses in Multiple Myeloma. J. Clin. Oncol..

[74] Harrison S.J., Minnema M.C., Lee H.C., Spencer A., Kapoor P., Madduri D., Larsen J., Ailawadhi S., Kaufman J.L., Raab M.S. (2020). A Phase 1 First in Human (FIH) Study of AMG 701, an Anti-B-Cell Maturation Antigen (BCMA) Half-Life Extended (HLE) BiTE^®^ (bispecific T-cell engager) Molecule, in Relapsed/Refractory (RR) Multiple Myeloma (MM). Blood.

[75] Bladé J., Miguel J.F.S., Fontanillas M., Esteve J., Maldonado J., Alcala A., Brunet S., Garcia-Conde J., Besalduch J., Moro M.J. (2001). Increased conventional chemotherapy does not improve survival in multiple myeloma: Long-term results of two PETHEMA trials including 914 patients. Hematol. J..

[76] Shimabukuro-Vornhagen A., Gödel P., Subklewe M., Stemmler H.J., Schlößer H.A., Schlaak M., Kochanek M., Böll B., Von Bergwelt-Baildon M.S. (2018). Cytokine release syndrome. J. Immunother. Cancer.

[77] Chen H., Wang F., Zhang P., Zhang Y., Chen Y., Fan X., Cao X., Liu J., Yang Y., Wang B. (2019). Management of cytokine release syndrome related to CAR-T cell therapy. Front. Med..

[78] Nooka A.K., Kastritis E., Dimopoulos M.A., Lonial S. (2015). Treatment options for relapsed and refractory multiple myeloma. Blood.

[79] Nijhof I.S., van de Donk N.W.C.J., Zweegman S., Lokhorst H.M. (2017). Current and New Therapeutic Strategies for Relapsed and Refractory Multiple Myeloma: An Update. Drugs.

